# Historical Perspective: How the Discovery of HPV Virus Led to the Utilization of a Robot

**DOI:** 10.3389/froh.2022.912861

**Published:** 2022-05-06

**Authors:** Grégoire B. Morand, Khalil Sultanem, Marco A. Mascarella, Michael P. Hier, Alex M. Mlynarek

**Affiliations:** ^1^Department of Otolaryngology Head and Neck Surgery, Centre Intégré Universitaire de Santé et de Services Sociaux West-Central Montreal-Jewish General Hospital, McGill University, Montreal, QC, Canada; ^2^Department of Radiation Oncology, Centre Intégré Universitaire de Santé et de Services Sociaux West-Central Montreal-Jewish General Hospital, McGill University, Montreal, QC, Canada

**Keywords:** radiotherapy, intensity-modulated, quality of life, oropharyngeal neoplasms, xerostomia

## Abstract

The treatment of oropharyngeal cancer has undergone many paradigms shifts in recent decades. First considered a surgical disease, improvements in radiotherapy led to its popularization in the 1990s. Subsequently, the discovery of the human papillomavirus (HPV) in the pathogenesis of oropharyngeal cancer, as well as the increase in HPV-associated oropharynx cancer incidence, have prompted a reevaluation of its management. Its sensitivity to standard treatment with a favorable prognosis compared to non HPV-associated oropharyngeal cancer led to a focus on minimizing treatment toxicity. Advances in radiation and surgical techniques, including the use of transoral robotic surgery, gave the rationale to ongoing de-escalation clinical trials in HPV-associated oropharynx cancer.

## Introduction

Similar to oral cavity cancer, oropharyngeal cancer has classically been addressed surgically with wide local excision of the tumor with margins and neck dissection to address the lymphatic drainage [1]. Unlike oral cavity cancer, some oropharyngeal tumors are difficult to access through the transoral route. These are approached using a mandibular swing, midline glossotomy, lateral pharyngotomy, or pull-through [2]. The morbidity and potential complications associated with these approaches are significant, resulting in swallowing difficulties, malocclusion following mandibular osteotomy, and wound complications. Additionally, the lower lip sometimes has to be split for access, leaving a visible scar on the face. Furthermore, damage to the hypoglossal, lingual, and superior laryngeal nerves is possible, which makes postoperative swallowing rehabilitation more challenging. In addition, depending on the extent of the resection, reconstruction with a vascularized free tissue flap is often necessary [2, 3].

The morbid surgical approach to oropharyngeal cancers led to the popularization of radiotherapy as the primary treatment modality [4]. With the exception of early-stage cancer (T1, small T2 with limited nodal disease), this is usually combined with concomitant chemotherapy [5]. Radiotherapy has several advantages: oropharynx tumors are generally radiosensitive and radiotherapy treats the primary tumor and the neck during the same setting [6]. In the case of larger tumors of the oropharynx that extend over several sub-regions and/or extend to neighboring areas, radiation therapy offers the advantage of treating and preserving the pharynx and larynx and potentially preserving swallowing function. Finally, advanced carcinomas of the tonsil or soft palate sometimes metastasize to the retropharyngeal lymph nodes (Rouvière), which is not so easily accessible surgically [7, 8].

Radiotherapy, however, also has treatment-related toxicities, such as xerostomia, dysphagia, changes in taste, and dental caries [9]. These effects are chronic and lifelong, which can have a major impact on the quality of life of patients who survive oropharyngeal cancer [9, 10]. Radiotherapy gained popularity during the 1990s as an alternative to surgery. The introduction of conformal radiotherapy (IMRT: intensity modulated radiotherapy), which minimizes radiation to surrounding normal tissue has decreased toxicity while maintaining treatment efficacy and further contributed to the standard use of this modality [11].

## Discovery of The Role of The Human Papilloma Virus In Oropharyngeal Cancer

Oropharyngeal carcinoma, like most head and neck squamous cell carcinomas, are classically caused by chronic exposure to extrinsic carcinogens such as cigarette smoke and alcohol. Thus, a patient who regularly smokes cigarettes is exposed to a higher risk of cancer of the mouth, oropharynx, larynx, and hypopharynx. However, a proportion of head and neck cancers occur in people without a history of tobacco or alcohol consumption, which suggests the contribution of other etiologies. Some epidemiological studies from the 1990s have shown that the incidence of cancer of the oropharynx was higher in geographical areas with high rates of cancer of the cervix [12, 13]. Since cervical cancer is caused by the human papillomavirus (HPV), this led to the hypothesis that a proportion of oropharyngeal cancers could also be caused by HPV [14, 15]. Many subsequent studies were able to confirm this hypothesis and demonstrate the causal link between HPV and a proportion of oropharyngeal cancer [16, 17].

Human papillomaviruses (HPVs) are DNA viruses that infect cells of the mucosal epithelium and are subsequently integrated into the cellular genome [18]. There are many HPV virus subtypes, but HPV serotypes 16, 18, 31, and 33 are considered the main carcinogenic serotypes. Transmission of the virus occurs from human to human through direct sexual contact [18]. The presence of the virus in tumor cells can be confirmed by the presence of HPV DNA by PCR or by *in situ* hybridization. In routine clinical practice, the presence of the protein p16, which is produced by cancer cells infected with HPV but cancer cells induced by extrinsic carcinogen exposure is often used as a surrogate marker for infection with HPV [18].

HPV-associated oropharyngeal cancer most commonly arises in the palatine tonsils (about 70% of cases), followed by base of tongue tonsillar tissue. For the other sites of the oropharynx, and for the oral cavity, larynx and hypopharynx, the role of HPV in oncogenesis is disputed, with likely <5% of cases attributable to HPV [19]. The predilection of HPV for the palatine and tongue base tonsils is poorly understood, but HPV is thought to infect the tonsil crypts where it finds an epithelium more susceptible to the transformative effects of the virus. For cervical cancer, HPV attacks the transformation zone between the squamous epithelium of the vagina and the columnar epithelium of the uterus [20].

## Implications of Human Papilloma Virus In The Treatment Of Oropharyngeal Cancer

During the 2000s, many studies showed an increase in the proportion of oropharyngeal cancers caused by HPV. The percentage of HPV-associated oropharyngeal cancer was around 15% in the 1990s. In 2020 this proportion was estimated to reach 80% in North America [21]. At the same time, it was also found that HPV-associated tumors had a better prognosis than non-HPV-associated tumors regardless of the treatment modality [22]. This means that if you irradiate or operate on a tumor of the oropharynx, the prognosis is better if it is positive for HPV [21].

HPV-associated oropharynx cancer usually occurs in younger and healthier patients, and has a very high probability of achieving a long term cure [21, 23]. Since the traditional treatments for oropharyngeal cancer are subject to substantial morbidity and side effects that can persist in the long term, clinicians have tried to de-intensify the treatment [24]. The objective is to achieve the same oncological outcome while diminishing the side effects of the treatment. The goal is to improve the long term quality of life and survivorship in patients with HPV-associated oropharynx cancer. It is important to emphasize that an attempt to de-escalate the treatment can only be made in the context of a clinical trial ensuring that the de-escalation of the treatment is not detrimental to oncological outcomes of the patients [25].

## Robotic Transoral Surgery As A Surgical De-Escalation Tool

Patients with HPV-associated cancer do better regardless of treatment modality. Beyond strategies aimed at limiting the toxicities related to radiotherapy, the possibility of using surgery as a primary modality has also been explored. In the early 2000s, technological progress allowed the popularization of the Da Vinci robot (Intuitive Surgical ©), which, due to its configuration, allows the surgeon to access the base of the tongue and the tonsils via a transoral route, thus avoiding the more morbid traditional external approaches to the oropharynx [26].

It is very important to understand the difference between non-surgical and surgical robots. A surgical robot is generally defined as a master-slave system where the machine is capable of performing movements directed by the surgeon. For transoral robotic surgery (TORS), the robot is merely an instrument that affords better visualization and access to remote areas of the oropharynx but is controlled by the surgeon. Therefore, it is more accurately called robot-assisted surgery.

Recent data suggests that the oncological results of robotic surgery are comparable to those of open surgery and chemoradiotherapy [27]. The ORATOR 1 study compared radiation vs. TORS in the treatment of early oropharyngeal cancers. Both modalities seemed to result in similar oncological outcomes but somewhat different side effect profiles [24]. Recently, the Eastern Cooperative Oncology Group (ECOG) 3,311 showed that upfront TORS followed by reduced doses of adjuvant radiation allows for similar oncologic results to standard chemoradiation in patients with locoregionally advanced HPV-associated oropharynx cancer [28].

Eventhou the benefits of TORS vs. radiotherapy are still controversial in early stage HPV-positive cancer [4], TORS seems to be a valid alternative to radiation therapy with similar oncologic outcomes and represents a de-escalation when compared to traditional surgical approaches. The most significant complication of robotic surgery is postoperative hemorrhage with an incidence rate ranging from 3 to 8% [24]. Although exceedingly rare, this complication can be fatal. The risk and amount of postoperative bleeding can be reduced by ligating branches of the external carotid artery [29].

A further disadvantage of TORS is the fact that postoperative radiation may be necessary is a significant proportion of cases, for which safe margins could not be achieved by surgery only [24]. Furthermore, postoperative radiation to the neck is often considered necessary for 2 or more positive nodes. In case of positive microscopic margins and/or extranodal extension, adjuvant chemoradiation is needed [30].

## Future Directions

There are several de-escalation strategies that have been performed for HPV-associated oropharynx cancers (Table 1). In 2019, two large randomized clinical trials evaluated a less toxic concomitant chemotherapy (cetuximab) demonstrated that standard therapy with concomitant high-dose cisplatin had a better cure rate and therefore remained the standard of care [36, 37]. However, interest in de-escalation has not waned and many more studies are underway.

**Table 1:** Deintensification strategies in HPV-positive oropharyngeal cancer.

**Strategy**	**Rationale**	**Study example**
Switch to less toxic concomitant chemotherapy	Novels agents such as PDL1 inhibitors or targeted therapy are potentially less toxic than traditional cisplatin	HN5 [31]
		REACH [32]
Radiotherapy dose reduction	Traditionally, primary tumor receives 70Gy, 66Gy/62Gy enough?	HN2 [33]
Surgical staging of the neck	Better risk stratification (extranodal extension, number of nodes).	PATHOS [34]
Neoadjuvant chemotherapy before surgery	Tumor load reduction before surgery.	NECTORS [35]
Tumor specific vaccination	Boosting of host specific immune reaction	NCT04369937

After several trials have shown the superiority of check-point inhibitors over standard chemotherapy in the recurrent and metastatic setting [38], numerous studies have tried to imitate those success in the definitive setting and to potentially deescalate the treatment for patients. Among the first studies with check point inhibitors in the definitive setting, the JAVELIN 100 study was negative as it failed to show a survival advantage in patients receiving avelumab compared to placebo in addition to standard chemoradiation [39, 40]. The interest for immunotherapy has however not faded. Trials similar to JAVELIN 100 in lung and esophageal cancer showed that the addition of check-point inhibitor in the *adjuvant* rather than *concomitant* setting let to survival improvement, suggesting that timing of stimulation of the immune system is critical to the oncologic outcome [39].

Given that standard chemoradiation treatment for patients with locoregionally advanced HPV-associated oropharynx cancer often leads to lifelong treatment-related toxicities and about 20% failure rate alternative treatment strategies are being sought. Our group has been evaluating the use of neoadjuvant platinum-based chemotherapy followed by robotic surgery for locoregionally advanced HPV-associated oropharyngeal cancer [American Joint Commission on Cancer version-7 (AJCC-7) Stage III (T1N1, T2N1, T3N0, T3N1) and stage IVa (T1N2, T2N2, T3N2)]. The Neoadjuvant Chemotherapy and Transoral Robotic Surgery for Oropharyngeal Cancer (NECTORS) study hypothesizes that treatment with neoadjuvant chemotherapy followed by transoral surgery and neck dissection is highly effective treatment allowing competitive cure rate compared to concurrent chemoradiotherapy with <10% failure rate, while avoiding radiotherapy in majority of cases. It is also hypothesized that better functional and quality of life outcome can achieved with this approach [41].

## Conclusion And Outlook

In conclusion, this review presents a brief history of oropharyngeal cancer treatment. Traditionally approached by morbid surgeries, advances in radiotherapy and the good response of oropharyngeal cancer to this treatment modality led to a paradigm shift. Subsequently, the discovery of the role of HPV in the oncogenesis of oropharyngeal carcinoma and the favorable prognosis associated with younger age at diagnosis have provided the rationale for de-escalation therapy for which many approaches are being evaluated. Among those, transoral robotic surgery (TORS) is a validated approach with similar outcomes to conformational radiotherapy in terms of tumor control and quality of life. Many trials are on-going to determine the effectiveness and side effects of different de-escalation strategies.

## Data Availability Statement

The original contributions presented in the study are included in the article/supplementary material, further inquiries can be directed to the corresponding author.

## Author Contributions

Study idea by AM. Manuscript drafting, literature review, and table by GM. All authors reviewed, amended, and approved the final version of the manuscript.

## Conflict of Interest

The authors declare that the research was conducted in the absence of any commercial or financial relationships that could be construed as a potential conflict of interest.

## Publisher's Note

All claims expressed in this article are solely those of the authors and do not necessarily represent those of their affiliated organizations, or those of the publisher, the editors and the reviewers. Any product that may be evaluated in this article, or claim that may be made by its manufacturer, is not guaranteed or endorsed by the publisher.
